# The Use of CD200 in the Differential Diagnosis of B-Cell Lymphoproliferative Disorders

**DOI:** 10.3389/bjbs.2023.11573

**Published:** 2023-09-26

**Authors:** Hanaan Kareem Al-Zubaidi, Stephen Fôn Hughes

**Affiliations:** ^1^ Pathology Department, Ysbyty Gwynedd, Betsi Cadwaladr University Health Board, Bangor, United Kingdom; ^2^ Maelor Academic Unite (MAU), Betsi Cadwaladr University Health Board, Wrexham, United Kingdom

**Keywords:** immunophenotyping, flow cytometry, chronic lymphocytic leukaemia, mantle cell lymphoma, hairy cell leukaemia

## Abstract

**Background:** B-Cell Lymphoproliferative Disorders (B-LPDs) are a group of heterogenous disorders characterised by the accumulation of B-cells in peripheral blood, bone marrow, lymph nodes and spleen. They have a variable disease course and outcome and many share similar features making differential diagnosis challenging. Therefore, accurate diagnosis is fundamental in particular for determining treatment options. Immunophenotyping by flow cytometry plays a crucial role in the diagnosis of B-LPDs. However, overlapping immunophenotyping patterns exist and the use of novel monoclonal antibodies has become increasingly important in immunophenotyping analysis. More recently differential expression of CD200 has been reported in various B-LPDs and that CD200 may improve the differentiation between chronic lymphocytic leukaemia (CLL) and mantle cell lymphoma (MCL). In this study CD200 expression is evaluated in different B-LPDs.

**Methods:** A total of 100 samples were collected and analysed by immunophenotyping flow cytometry over a period of 1 year (2017–2018), by a panel of monoclonal antibodies including CD200. The percentage of CD200 and its expression intensity was evaluated and compared between different groups of B-LPDs.

**Results:** All of the 50 cases of CLL expressed CD200 with moderate to bright intensity, 6 MCL cases lacked the expression of CD200. Furthermore, all 5 cases of hairy cell leukaemia (HCL) expressed CD200. Out of all B-LPDs evaluated, CD200 expression in HCL cases was noted to be the brightest. The other 39 cases were not found to be B-LPDs.

**Conclusion:** CD200 has an important role in differentiating CLL from MCL, HCL has a consistent bright expression of CD200. By adding CD200 to the combinations of markers in routine testing panel, Immunophenotyping by flow cytometry can be an effective tool in the diagnosis of B-LPDs especially in cases with atypical immunophenotyping pattern. Our result support that CD200 can be added to routine testing panel as it is useful in differentiating them.

## Introduction

B-Cell Lymphoproliferative disorders (B-LPDs) consist of a wide range of heterogenous leukaemias and lymphomas characterised by the proliferation of mature B lymphocytes in the peripheral blood, bone marrow and lymphoid tissues [1]. The World Health Organization (WHO) 2017 classification of tumours of haematopoietic and lymphoid neoplasms has classified B-LPDs into different subtypes and their diagnosis is achieved using a multisystem approach based on morphology, immunophenotyping, molecular biology features and cytogenetics [2]. However, there is a significant overlap in some B-LPDs, in particular in their clinical presentation and similar morphological appearance of B cells seen in many. In addition, their clinical outcome differs significantly in terms of treatment options and survival rates. Therefore, an accurate diagnosis is essential and the use of immunophenotyping analysis has become an increasingly important method that is widely used in haematology. Out of B-LPDs, chronic lymphocytic leukaemia (CLL) is the most common leukaemia in Europe and North America [3]. CLL is strongly associated with age and higher incidences are seen in males. There were 3,709 new cases of CLL diagnosed in the UK in 2015 [4].

In 1994 Matutes et al developed a scoring system for the diagnosis of CLL which is based on the immunophenotyping analysis of five markers CD5, CD23, FMC7, CD79b/CD22, and surface membrane immunoglobulin (SmIg). CLL is usually diagnosed when the circulating B lymphocytes exhibit the characteristic immunophenotyping pattern of CD5^+^, CD23^+^, FMC7-, CD79b-/CD22- and a weak expression of surface membrane immunoglobulin (SmIg). One score is given for each marker and typical CLL cases are easily identified by a ≥4/5 score. However, problems arise when diagnosing cases that exhibit a non-characteristic immunophenotyping pattern such as atypical CLL cases with a 3/5 score and those non-CLL cases with a 0–2/5 score [5]. Therefore, differential diagnosis between CLL and other B-LPDs remains a big challenge [6], with some studies proposing a new differential diagnosis algorithm [7]. Thus, new and novel markers are continuously required for immunophenotyping analysis [8]. More recently, it has been discovered that CD200 has a differential expression in B-LPDs such as CLL and MCL which indicated a possible diagnostic value [9].

CD200 (previously known as MRC OX-2) is a membrane glycoprotein belonging to the immunoglobulin superfamily [10], it is encoded by a gene located on chromosome 3q12 [11]. CD200 is expressed on a variety of cell types, including myeloid cells, dendritic cells, neurons, endothelial cells, as well as B and T-lymphocytes [12]. The widely expressed CD200 interacts with the CD200 receptor (CD200R) an inhibitory receptor expressed on monocytes, neutrophils, basophils, macrophages and dendritic cells [13, 14] that plays a vital role in regulating an immune response [15, 16].

The aim of this study was to evaluate the expression of CD200 in various B-LPDs, to determine its usefulness in its differential diagnosis properties in cases already tested at Haematology laboratory, Ysbyty Gwynedd. To support its use in routine Immunophenotyping testing panels. This is a retrospective study, which is part of a service evaluation project.

## Materials and Methods

Cases tested for lymphoproliferative disease screen at the Specialised Haematology laboratory within the blood sciences department in Ysbyty Gwynedd Hospital between 2017 and 2018 were included in this study. The specialised haematology service in Ysbyty Gwynedd, Bangor is the only service which provides diagnostic Immunophenotyping by flow cytometry analysis for North Wales, covering three hospital sites (Ysbyty Gwynedd in Bangor, Ysbyty Glan Clwyd in Rhyl and Ysbyty Wrexham Maelor in Wrexham). Most samples are received from Haematology and oncology services within the three hospital sites to confirm the diagnoses of haematological disorders. A total of 100 peripheral blood samples were already analysed at the time of diagnostic request and their data were retrospectively evaluated. The B-LPDs included are CLL (*n* = 50), MCL (*n* = 6), HCL (*n* = 5), and other (*n* = 39).

Immunophenotyping test is part of the standard diagnostic work up requested by consultant haematologists and GPs that is required to make an accurate haematological diagnosis in which all cases were diagnosed in accordance with the WHO 2017 classification [17] which is based on clinical, morphological and immunophenotyping analysis [2]. The Matutes score was calculated in all cases [18].

This study was approved by the Clinical Effectiveness Department, Ysbyty Gwynedd, Betsi Cadwaladr University Health Board NHS Trust and the Faculty of Medicine, Dentistry and Life Sciences Research Ethics Committee, University of Chester (FREC reference: 1446(1416)/18/HAZ/CMS) [19].

Although this was a retrospective data analysis study on pre-collected and pre-analysed samples. The procedure that was used for analysis is described below.

### Flow Cytometry Immunophenotyping Analysis

An Immunophenotyping panel was performed on peripheral blood (PB) samples, using a combination of fluorescein isothiocyanate (FITC), phycoerythrin (PE), phycoerythrin-texas red (ECD), phycoerythin cyanin 5 (PC5), phycoerythrin cynanin 7 (PC7) fluorescent conjugated monoclonal antibodies (MoAb). MoAbs used were sourced from Beckman Coulter, France.

First a full blood count (FBC) was performed on all samples to check that the WBC was <5 × 10^9^ on Sysmex XE5000 analyser. A total of 100 µL of EDTA peripheral blood sample was incubated for 15 min in the dark with 10 µL MoAbs. The routine immunophenotyping testing panel in our haematology department for the diagnosis of B-LPDs on PB or BM includes a four-colour combination of MoAbs for CD45, CD2, CD5, CD10, CD19, CD20, CD22, CD23, FMC7, CD43, CD11c, CD103, CD25, CD16/56, Kappa and lambda light chains. In addition, CD200 was included in all cases. A tube containing Ig specific isotype controls for FITC/PE/PC5/ECD were used in all cases, and staining was obtained using the lysed-wash technique, lysed with ammonium chloride lysing buffer and washed in PBS. Tubes were resuspended in PBS, vortexed and analysed using the Beckman Coulter FC500 flow cytometer. Daily quality control procedures were performed using Flow-check and Flow-set beads according to the laboratory standard operating procedures to verify consistent fluorescence intensity during the study.

After completion, data acquisition was performed immediately. For each sample, data from at least 5 × 10^3^ events per tube was acquired. Gating on lymphocytes was achieved on CD45 versus side scatter analysis.

CD200 expression was evaluated by comparison with isotype control and the antigen expression was defined as positive according to the flow cytometric immunophenotyping consensus guidelines (AIEOP-BFM) [20]. CD200 expression intensity was categorised as negative, dim, moderate, and bright.

**FIGURE 1:**
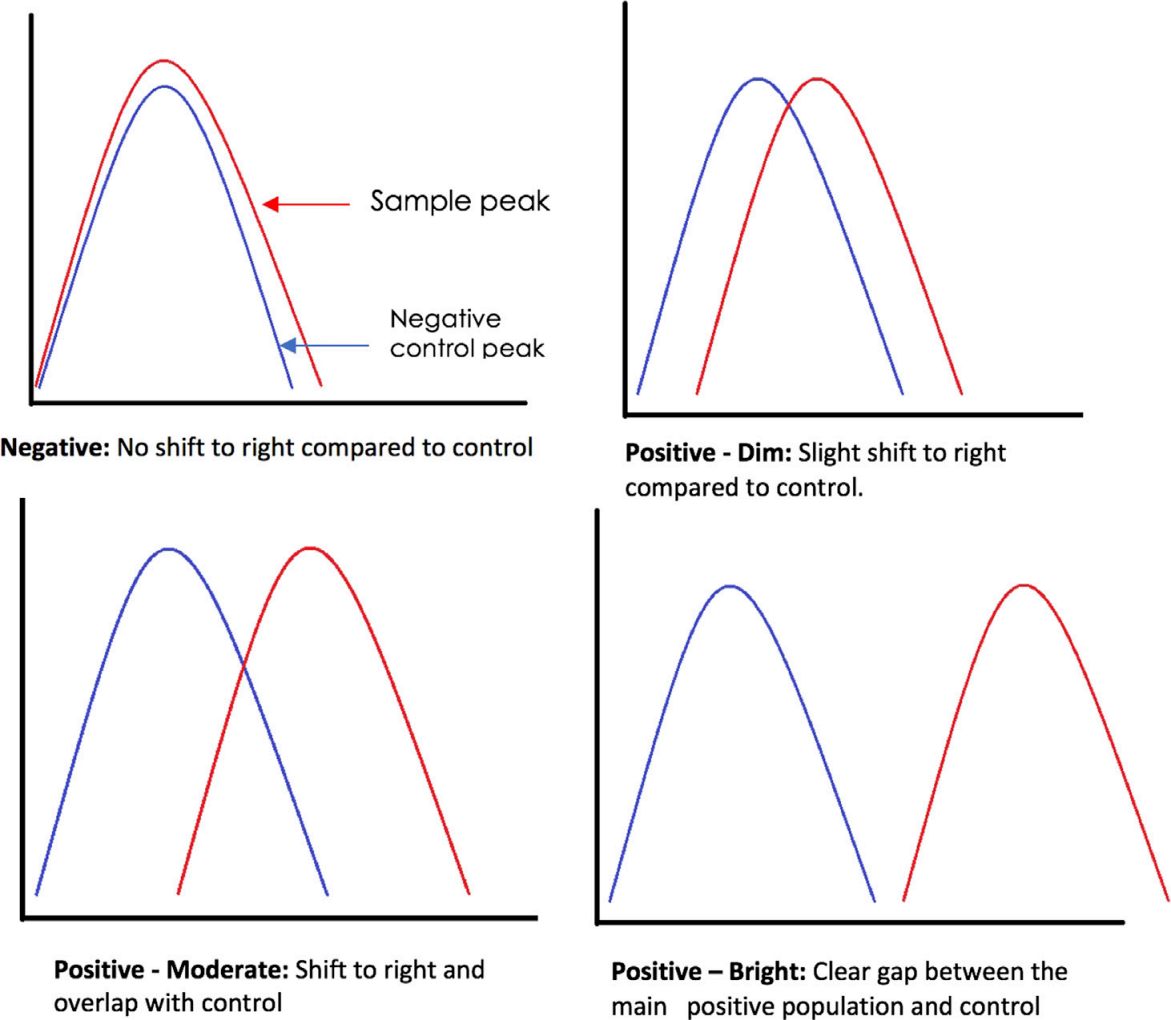
Flow cytometry antigen expression interpretation.

**FIGURE 2:**
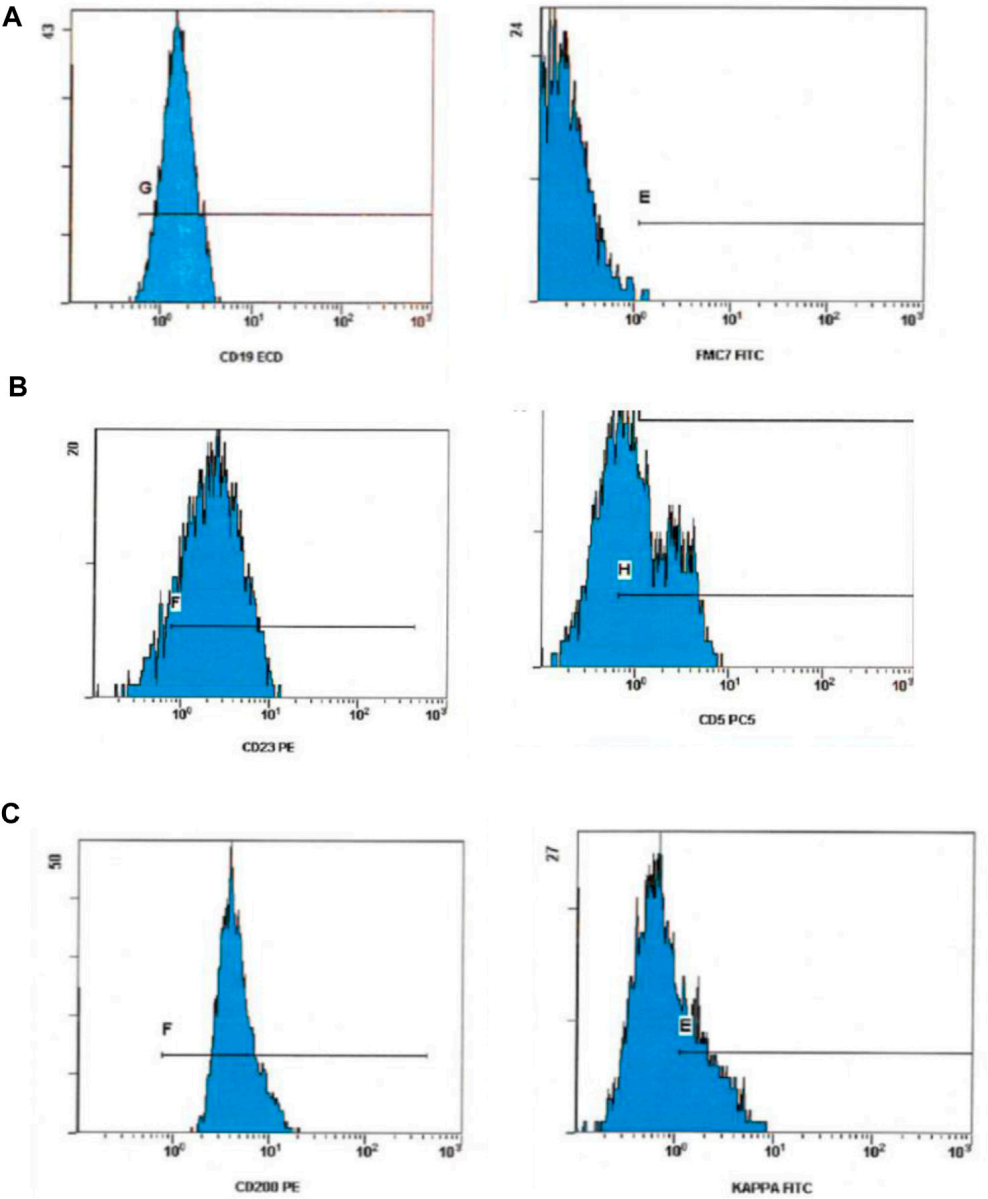
Flow cytometry scatter plot showing expression pattern in CLL [Row **(A)**] CLL with a negative FMC7, [Row **(B)**] positive CD23 and CD5 expression. And, [Row **(C)**] a bright CD200 and dim kappa ex-pression.

CD200 is negative when there is no shift to right compared to isotype control tube, dim expression is when there is a slight shift in peak compared to negative control, moderate is when the peak shifts to right and overlaps with the negative control. And bright is where there is a clear gap between the main positive population and the negative control with no virtual overlap (Figure 1). An example of scatter plot showing expression seen in CLL is shown in Figure 2.

### Statistical Analysis

Data collected were analysed with the appropriate statistical tests. Descriptive statistics was presented as number of cases, percentages, Means and SD were calculated. Comparison between groups was performed using non-parametric one-way ANOVA test. All statistical tests were carried out using the statistical package SPSS for Mac version 23 (SPSS Inc., Chicago, IL, USA). Statistical difference was defined when the *p*-value less than 0.05.

## Results

**FIGURE 3:**
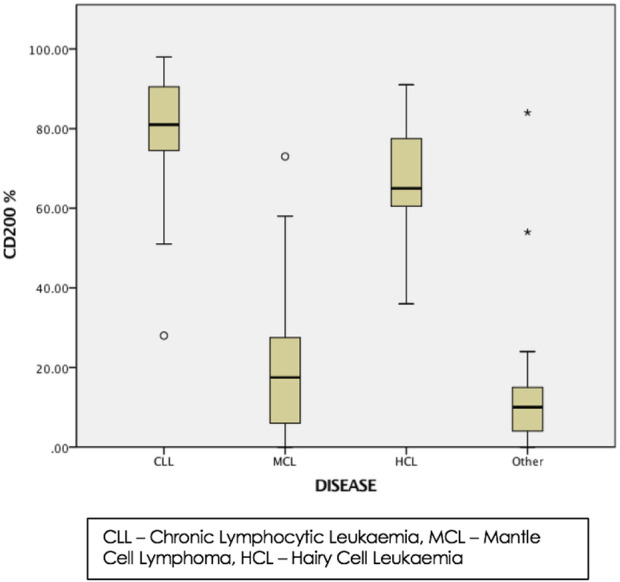
Box plot showing the % of cells with positive expression for CD200 in B-LPDs. The lines inside the box and bars represent median and interquartile ranges. Asterisks and circles represent CD200 values not included between the vertical lines (outliners).

Data from 100 cases tested by flow cytometry Immunophenotyping was retrospectively evaluated. Out of the 100 cases, CLL accounted for the majority of cases, comprising of 50 (50%) cases, of which there were 35 males and 15 females with median age 72µyears. The median haemoglobin (Hb), total white blood count (WBC), lymphocytes count (Lymphs), and platelet count (Plt) were 133.5 g/L, 18 × 10^9^/L, 12 × 10^9^/L, and 209.5 × 10^9^/L respectively. MCL accounted for 6 (6%) of cases, there were 3 males and 3 females with median age 75 years. The median Hb, WBC, Lymphs and Plt count were 126 g/L, 14.8 × 10^9^/L, 9.9 × 10^9^/L, and 136.5 × 10^9^/L respectively. HCL accounted for 5 (5%) cases, of which there were 2 males and 3 females with median age of 56 years. The median Hb, WBC, Lymphs and Plts counts were 126 g/L, 8.3 × 10^9^/L, 1.1 × 10^9^/L, and 155 × 10^9^/L respectively. There were 39 other cases which were found not to have B-LPDs and were therefore, not discussed (see Tables 1, 2; Figure 3). The percentage of CD200 positive cells was compared between the three groups, there was a significant statistical difference in % of CD200 between CLL and MCL (*p* < 0.001), and between HCL and MCL (*p* < 0.001). There was no statistical difference in the percentage of CD200 between CLL and HCL (*p* > 0.05.)

**TABLE 1:** CD200 expression pattern and percentage of positive cells in B-LPDs by Immunophenotyping Flow Cytometry Analysis.

Case	CD200 expression	No. of cases	% Of cells with positive expression of CD200 (mean, median, SD)	Pattern of CD200 expression
CLL	Positive	50	79.9, 81, 13.4	Moderate to Bright
	Negative	0	—	
MCL	Positive	0	—	Absent to dim
	Negative	6	10, 10.5, 9.6	
HCL	Positive	5	64.5, 65, 20.1	Bright
	Negative	0	—	

CLL, chronic lymphocytic leukaemia; MCL, mantle cell lymphoma; HCL, hairy cell leukaemia.

**TABLE 2:** Clinical characteristics of CLL, MCL and HCL patients.

	CLL	MCL	HCL
Age (Mean, Median, SD)	70, 72, 10.5	72.5, 75, 6.8	59.6, 56, 12.4
Sex M/F	35/15	3/3	2/3
Total	50	6	5
Haemoglobin (Mean, Median, SD)	131, 133.5, 21	124.5, 126, 8.9	124, 126, 5.8
White Blood cells (Mean, Median, SD)	31.7, 18, 39	21, 14.8, 15	6.3, 8.3, 3.6
Lymphocytes (Mean, Median, SD)	25, 12, 38	15.9, 9.9, 13.8	2.1, 1.1, 2.5
Platelets (Mean, Median, SD)	220, 209.5, 103	149.8, 136.5, 64.9	153, 155, 70

CLL, chronic lymphocytic leukaemia; MCL, mantle cell lymphoma; HCL, hairy cell leukaemia.

## Discussion

Studies have reported that CD200 is expressed in haematological malignancies such as multiple myeloma (MM), acute myeloid leukaemia (AML) and acute lymphoblastic leukaemia (ALL) [21, 22]. In MM and AML, CD200 expression is used as a prognostic markers as high levels of expression are associated with poor prognosis [23, 24]. CD200 expression was then reported for CLL [22]. Since then, studies have shown that CD200 was expressed differently between CLL and mantle cell lymphoma (MCL) it is consistently expressed in CLL whereas MCL lack the expression of CD200 [9, 25–27]. In addition, CD200 is also expressed in hairy cell leukaemia (HCL) [28].

In this study, CD200 expression was retrospectively evaluated in samples pre-analysed as part of routine immunophenotyping testing at Ysbyty Gwynedd, Bangor, to validate its usefulness in differentiating between different B-LPDs.

### CD200 Expression in CLL

There is no single marker for the definitive diagnosis of CLL by Immunophenotyping and in general, the diagnosis of CLL is easily achieved in the presence of the characteristic immunophenotyping pattern (CD5^+^, CD23^+^, FMC7-, CD22-/CD79b- and weak expression of SmIg) which is based on the Matutes scoring system [29]. However, problems arise in both diagnosing and differentiating CLL from other B-LPDs in cases where immunophenotyping features are not typical such as cases with Matutes score of 2–3/5 as a study showed that in using this system, they found that 92% of CLL cases score 4 or 5, score 3 is seen in about 6% of CLL cases and 2% of CLL cases score 1 or 2 [30] which results in additional markers being analysed to aid in the diagnosis and further testing is usually the case to make a definitive diagnosis.

We have confirmed previous studies that CD200 is consistently expressed in CLL [9, 31-33], with 50/50 (100%) of CLL cases in this study expressing CD200 (mean % of cells with positive expression of CD200 = 79.9%). A similar finding was also reported where CD200 expression was observed in 100% of CLL in similar studies [9, 25, 34, 35].

### CD200 Expression in CLL and MCL

MCL cases in this study lacked CD200 expression. Confirming previous studies [9, 21, 25, 34]. The difference in CD200 expression between CLL and MCL cases was found to be statistically significant.

For many years, CD23 has been a reliable marker that is widely used to differentiate between B-LPDs, in particular CLL and MCL as it is positive in CLL and negative in MCL [36, 37]. However, CD23 as a single marker is not sufficient to make a definitive distinction between CLL and MCL. In addition, in occasional cases, it has been reported that CLL have weak or no expression of CD23 [26, 38], and a minority of MCL cases can express CD23 as studies have shown that approximately 20%–30% of MCL cases can have a positive CD23 expression [26, 39–41].

Other antigens that are used to distinguish between CLL and MCL include FMC7, which is usually negative in CLL and expressed in MCL [42]. However, similar to CD23, studies have reported positive expression of FMC7 is seen in about 12% of CLL cases [36, 43–45] and 90%–100% of MCL cases [36, 42], making cases with an unusual phenotype difficult to diagnose.

The differentiation between MCL and CLL is crucial as MCL is characterised by an aggressive clinical course with continuous relapse and poor prognostic outcome [46–48], and sometimes clinicians rely on other tests such as testing for Cyclin D1 and/or the detection of chromosomal translocation t(11; 14) for a definitive diagnosis which uses techniques such as polymerase chain reaction (PCR) and Western blot which are both costly, time consuming and not available in all Haematology laboratories. In addition, there have been reported cases of MCL which lack the positivity of Cyclin D1 [49, 50]. Therefore, we believe in cases were diagnosis is difficult to achieve, the addition of CD200 as an extra marker is useful in differentiating between CLL and MCL as Immunophenotyping by flow cytometry is not difficult, is fast and less expensive. In this study, CD200 was consistently expressed in CLL cases and MCL cases lacked the expression of CD200. CD200 was found to be excellent marker in differentiating between CLL and MCL.

### CD200 Expression in CLL and HCL

In this study, CD200 expression was observed in all HCL cases 100% (5/5) the mean % of cells with positive expression of CD200 = 64.5%, which was a similar finding reported in other studies [25, 35, 51]. Also, the HCL cases in this study showed the brightest intensity for CD200 expression out of all cases. Bright CD200 expression in HCL cases has also been reported in similar studies [25, 51–54]. Others have confirmed CD200 expression in HCL by Immunohistochemistry analysis on formalin fixed paraffin embedded tissue sections of bone marrow biopsy and lymph nodes [21].

The characteristic immunophenotyping feature of HCL is the expression of CD19, CD20, CD22, CD25, CD11c, CD103 and SmIg [17, 55, 56]. Also, to differentiate HCL from CLL, HCL is usually CD23 negative and FMC7 positive whereas CLL has the opposite phenotype [29]. Discrepancy in the classic immunophenotyping pattern in HCL has been described with one study demonstrated positive expression of CD23 in about 17% of HCL cases, the lack of CD25 expression in 3% of HCL cases, and lack of CD103 in 6% of HCL cases [55]. Additionally, the markers expressed in HCL such as CD25, CD103 and CD123 are often not present in the initial immunophenotyping testing panel for the diagnosis of LPDs, as such an additional panel using extra markers might have to be set up. By adding CD200 to the initial routine testing panel, the bright expression of CD200 might raise a suspicion that HCL is likely.

It has been reported that CD200 is not expressed in a variant form of HCL (HCLv) [28, 52]. Although no cases of HCLv was included in this study. HCLv has a similar clinical and morphological features to HCL [57, 58] but patients are resistant to HCL therapy and require special treatment options [59, 60]. Therefore, differentiating between the two is important.

CD200 not only aids in confirming a diagnosis of HCL, but also distinguishes HCL from HCLv. We believe that adding CD200 to the initial immunophenotyping testing panel would be significantly beneficial.

Overall, the findings of this study agree with previous studies confirming the expression of CD200 in CLL, HCL and its lack of expression in MCL. The expression of CD200 in other similar studies is summarised in Table 3.

**TABLE 3:** CD200 expression reported in similar studies.

		CD200 expression		
Study	Total No of cases	CLL cases	MCL cases	HCL cases
[35]	50	30/30 (100%)	—	5/5 (100%)
Poongodo R et al., 2018	77	54/54 (100%)	1/6 (16%)	5/5 (100%)
[28]	160	98/98 (100%)	0/24 (0%)	6/6 (100%)
Gorczynski et al., 2017	70	45/45 (100%)	0/14 (0%)	—
Mason et al., 2017	79	—	—	34/34 (100%)
Fan L et al., 2015	374	268/271 (98.8%)	1/31 (3%)	2/5 (40%)
El-Sewefy DA et al., 2014	40	30/30 (100%)	0/10 (0%)	—
[53]	159	56/56 (100%)	0/14 (0%)	13/13 (100%)
[54]	364	119/119 (100%)	3/61 (5%)	7/7 (100%)
[52]	180	27/27 (100%)	0/14 (0%)	10/10
[34]	107	19/19 (100%)	0/4 (0%)	—

CLL, chronic lymphocytic leukaemia; MCL, mantle cell lymphoma; HCL, hairy cell leukaemia.

This study has its limitation, first the sample size was relatively small limited to 100 samples, as this was a retrospective data analysis for samples tested over a 1 year period, this could be improved by extending the period for the retrospective collection of data for about two or three years. Also, during the 1 year, the cases diagnosed were limited to CLL, HCL and MCL so may be other B-LPDs would have been diagnosed if the study was extended to allow more cases to be included.

The majority of studies have focused on the use of CD200 to differentiate between CLL and MCL, limited reports on other B-LPDs are available. Thus, more studies are needed to include cases for other B-LPDs. In particular, it would be useful to analyse CD200 expression in those MCL cases that are negative for cyclin D1. Also, there are a limited number of reports which have evaluated the expression of CD200 between HCL and HCLv and used a small number of cases. Therefore, the role of CD200 in differentiating between HCL and HCLv could be investigated further. Although many have investigated CD200 expression by flow cytometry, some reports have also demonstrated its usefulness in immunohistochemistry [21]. Further studies comparing CD200 expression using the two methods could be beneficial.

In addition to its diagnostic value, CD200 has been shown to have a prognostic role in diseases such as in acute lymphoblastic leukaemia [61]. Its expression has been associated with an unfavourable prognostic outcome in AML and MM [62]. And in CLL, low expression of CD200 has been associated with predicting shorter time needed for treatment [63].

There has been some work into anti-CD200 targeted therapy and that anti-CD200 can supress tumour cells and restore tumour immune control in an animal model [64]. This has led to the development of humanised monoclonal anti-CD200 antibody ALXN600 used in phase I/II clinical trial (NCT00648739) for patients with CLL and MM with only mild to moderate side effects reported [65]. The overexpression of CD200 has also been involved in the pathogenesis of various tumours including renal, colon, testicular head and neck carcinoma [66–68]. Thus, targeting CD200 may be hopeful for the future in a large number of malignancies.

To conclude, there is no single marker for the definite diagnosis of CLL by Immunophenotyping and although the Matutes score has been the basis of the diagnosis of CLL, sometimes relying on the markers used in the score alone is not sufficient in the differential diagnosis with some cases relying on further testing such as Immunohistochemistry (IHC), cytogenetics fluorescence *in situ* hybridisation (FISH) testing which are time consuming, expensive and are not always available in all haematology laboratories and require sending to other centres with additional samples requested. Since Immunophenotyping testing is already performed for the diagnosis of B-LPDs cases, and the addition of further markers will not only be time saving but also economical as no additional diagnostic sample would be required. Therefore, the addition of an extra marker such as CD200 is of a significant diagnostic value.

Our results confirm that CD200 is a valuable marker in confirming the diagnosis of CLL and HCL, adding CD200 to the routine testing panel will aid in differentiating between CLL and MCL in cases with overlapping immunophenotyping results. Therefore, it should be included in routine testing Immunophenotyping panels to aid in differentiating between various B-LPDs. In the future, CD200 could be a possible therapeutic target especially in patients with a bright CD200 expression as seen in HCL and CLL.

## Summary Table

### What is Known About This Topic?

• There is no single marker for the definite diagnosis of B-cell Lymphoproliferative disorders.
• CD200 is expressed in B-cell Lymphoproliferative disorders such as CLL and HCL.

### What This Work Adds

• It expands the understanding of CD200 expression in B-cell Lymphoproliferative disorders.
• It confirms the importance of CD200 in diagnosing CLL and HCL as well as differentiating between various B-cell Lymphoproliferative disorders.
• It gives further support to include CD200 in routine Immunophenotyping testing panels to aid in the diagnosis of B-cell LPDs.

This work represents an advance in biomedical science because it shows the importance of CD200 and its significant diagnostic value when used in immunophenotyping panels for the diagnosis of B-cell Lymphoproliferative disorders.

## Data Availability

The original contributions presented in the study are included in the article/supplementary material, further inquiries can be directed to the corresponding author.

## Abbreviations

B-LPDs, B-cell lymphoproliferative disorders; CLL, chronic lymphocytic leukaemia; MCL, mantle cell lymphoma; HCL, hairy cell leukaemia; PB, peripheral blood; FITC, fluorescein isothiocyanate; PE, phycoerythrin; ECD, phycoerythrin-texas red; PC5, phycoerythin cynanin 5; PC7, phycoerythin cynanin 7; MoAbs, fluorescent conjugated monoclonal antibodies; FBC, full blood count; MM, multiple myeloma; AML, acute myeloid leukaemia; ALL, acute lymphoblastic leukaemia; HCLv, hairy cell leukaemia variant.
